# Supplementary material for: SON drives oncogenic RNA splicing in glioblastoma by regulating PTBP1/PTBP2 switching and RBFOX2 activity
Source: Nat Commun. 2021 Sep 21;12:5551. doi: 10.1038/s41467-021-25892-x (PMC8455679; doi:10.1038/s41467-021-25892-x)
Supplement: Supplementary file 1 — Supplementary Information [file 41467_2021_25892_MOESM1_ESM.pdf]

Supplementary Information

SON drives oncogenic RNA splicing in glioblastoma by regulating PTBP1/PTBP2 switching and RBFOX2 activity

Jung-Hyun Kim[*], Kyuho Jeong[*], Jianfeng Li[*], James M. Murphy, Lana Vukadin, Joshua K. Stone, Alexander Richard, Johnny Tran, G. Yancey Gillespie, Erik K. Flemington, Robert W. Sobol[**], Ssang-Teak Steve Lim[**] and Eun-Young Erin Ahn[**]

- SUPPLEMENTARY INFORMATION

Supplementary Figures 1 – 13

Supplementary Tables 1 and 2

Supplementary Figures

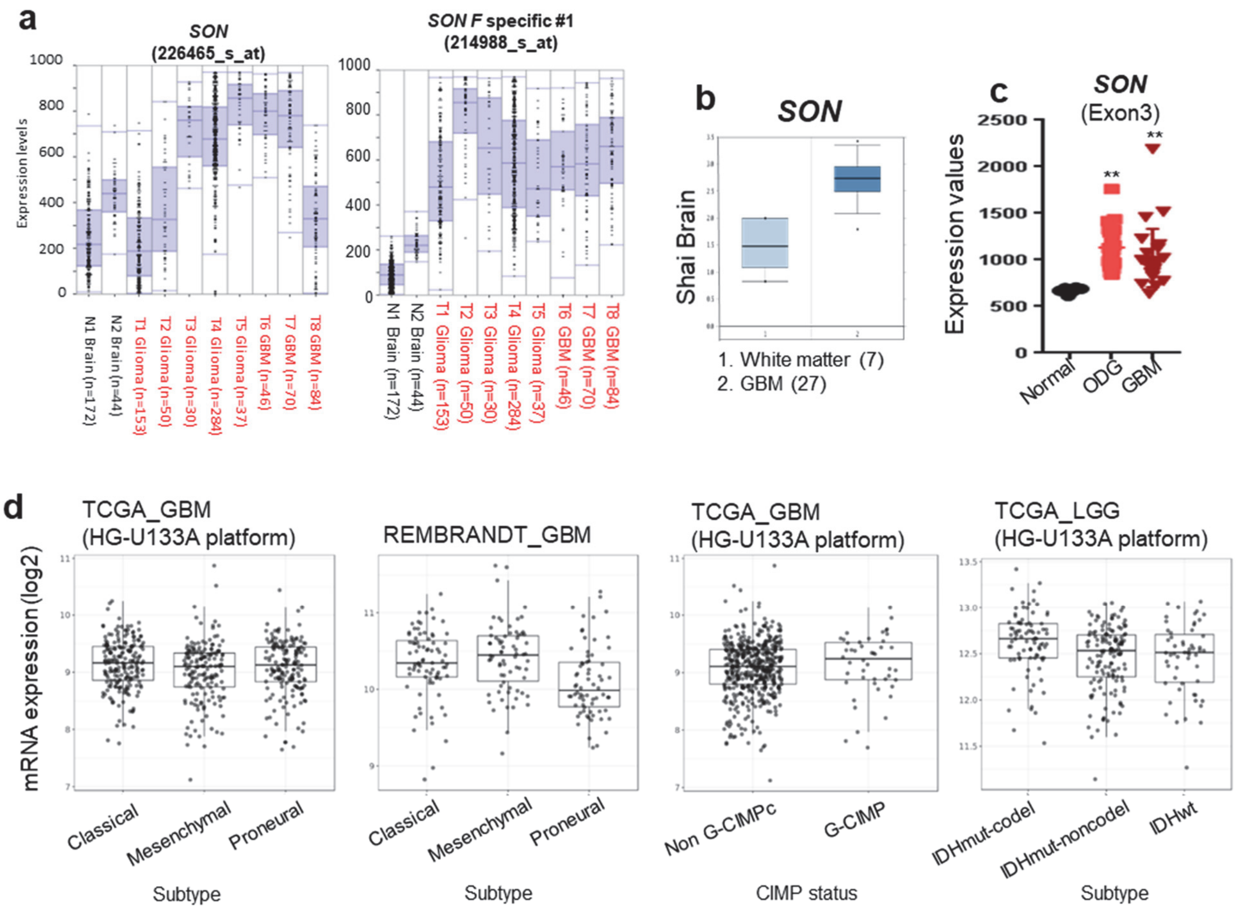

Supplementary Figure 1. SON is upregulated in brain tumor patients. a Analysis of brain tumor data sets from the R2 database for the mRNA level of *SON* (probe set 226465_s_at which targets exon 1 – exon 3 regions and probe set 214988_s_at which targets 3'UTR specific for SON F) in 5 glioma data sets, 3 glioblastoma (GBM) sets compared with 2 normal brain sets. $p<0.01$. **b** Relative expression levels of *SON* analyzed from Oncomine database. The values of log2 median-centered intensity were displayed as a boxplot according to Oncomine output. **c** *SON* expression from the Exon Expression Array dataset (GSE9385) for normal brain (n=6), ODG (oligodendroglioma; n=23) and GBM (glioblastoma; n=26). Error bars in all graphs represent standard deviation (SD) of tests. $^*p<0.05$, $^{**}p<0.01$. Statistical significance was determined by an unpaired two-tailed t-test. **d** Similar levels of SON expressions are detected in various subtypes of GBM and low grade glioma (LGG). SON mRNA expression levels in indicated subtypes of GBM or LGG were analyzed from TCGA and REMBRANDT datasets using the GlioVio data portal (http://gliovis.bioinfo.cnio.es/). *G-CIMP*, (glioma cytosine-

phosphate-guanine (CpG) island methylator phenotype; *IDHmut-codel* (hemizygous co-deletion of chromosome arms 1p/19q); *IDHmut-noncodel* (without co-deletion of 1p/19q) gliomas. Source data are provided in the Source data file.

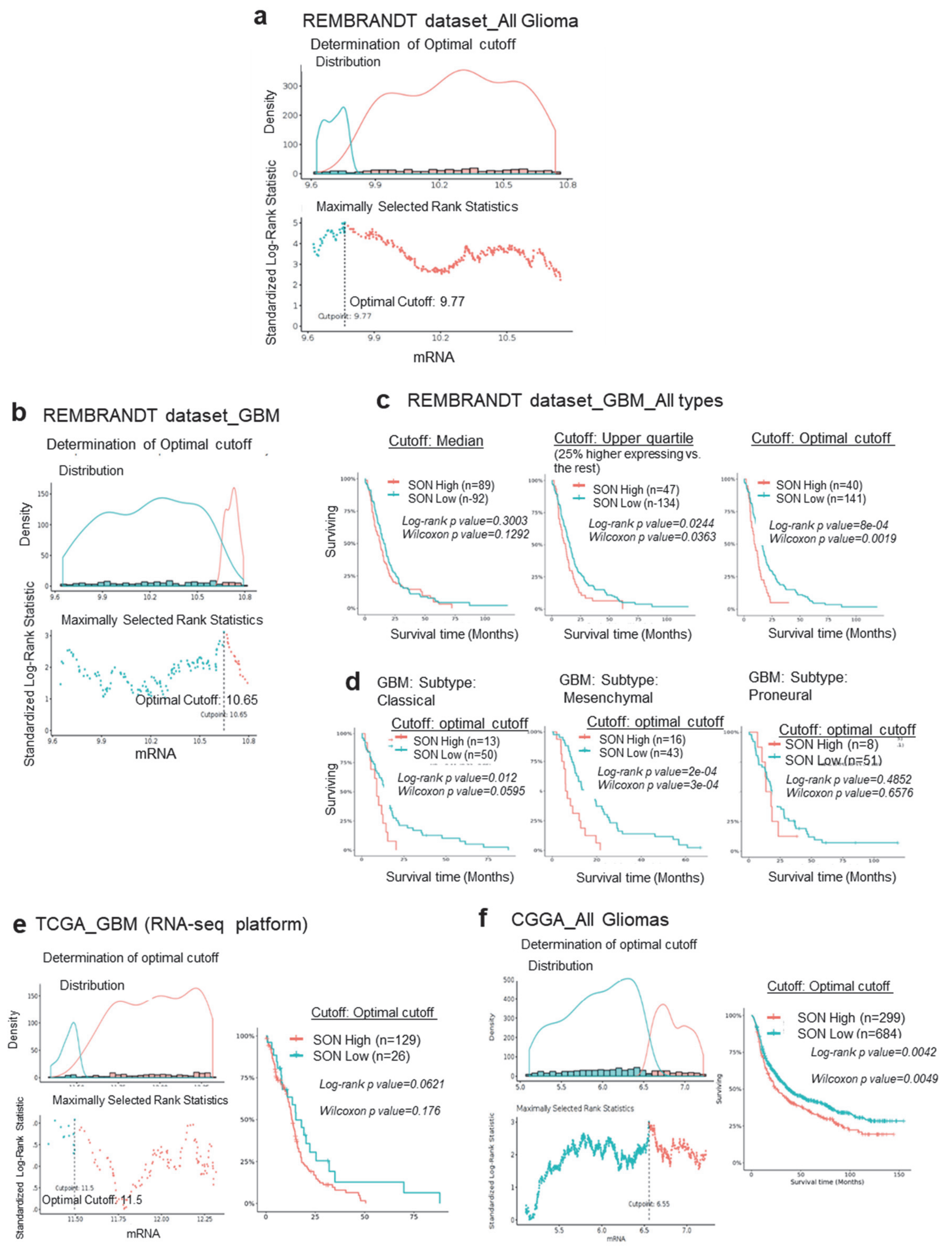

Supplementary Figure 2. High levels of SON correlate short survival of glioma patients.

a. Determination of optimal cutoff for REMBRANDT data set (All Glioma). The optimal cutoff

value was calculated using the maximally selected rank statistic and this value was used to divide SON-high and SON-low groups for a Kaplan–Meier overall survival curve presented in the **Figure 1d**. **b.** Determination of the optimal cutoff value for REMBRANDT data set (GBM only). **c.** Kaplan–Meier overall survival curves of patients from REMBRANDT GBM dataset (All types). Cutoff values dividing the SON-high and SON-low groups were median (left), upper quartile (middle), and optimal cutoff value (right) as indicated. **d** Kaplan–Meier overall survival curves of patients with different subtypes of GBM (classical, mesenchymal, proneural) from REMBRANDT GBM dataset. **e, f.** Determination of the optimal cutoff value for the TCGA_GBM (RNA-seq platform) dataset (**e**), and CGGA_All Glioma dataset (**f**) and the associated Kaplan–Meier overall survival curves with SON-high and SON-low groups determined by the optimal cutoff value. All analyses were performed using GlioVis data portal.

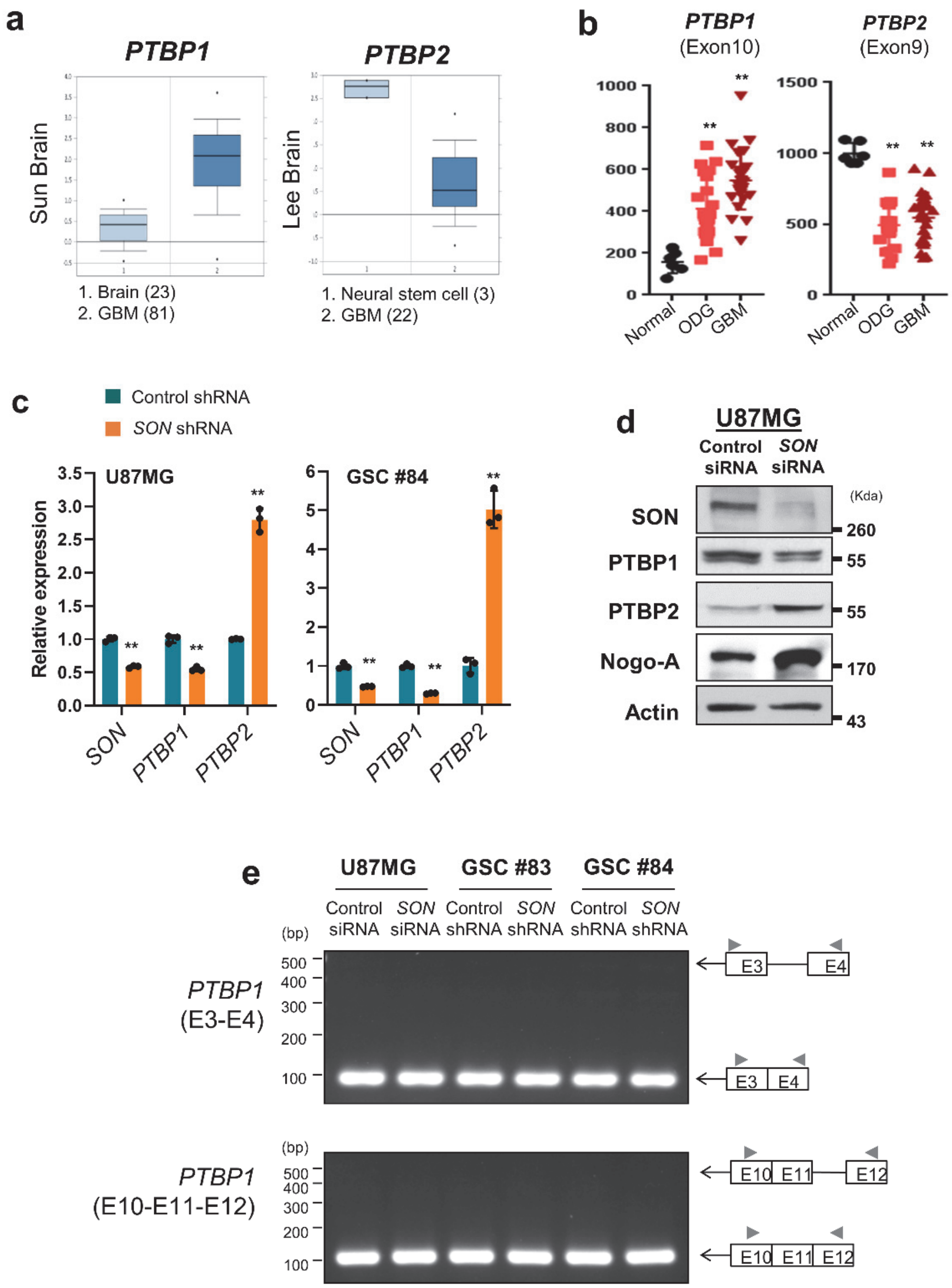

Supplementary Figure 3. SON knockdown leads to decreased PTBP1 expression while increasing PTBP2 expression. a. Relative expression levels of *PTBP1* and *PTBP2* analyzed from Oncomine database. The values of log2 median-centered intensity were displayed as a boxplot according to Oncomine output. **b.** *PTBP1* and *PTBP2* gene expression from the Exon Expression Array dataset (GSE9385) for normal brain (n=6), ODG (oligodendroglioma; n=23)

and GBM (glioblastoma; n=26). ** p< 0.01. **c, d.** The effect of SON depletion on expression of PTBP1 and PTBP2 in U87MG cells and GSC #84 (patient-derived glioma-stem cells) were measured by RT-qPCR (**c,** n=3) and Western blot analysis (**d**). WB Data are representative of n = 3 independent experiments. **e** No retention was observed in *PTBP1* introns 3, 10, and 11 upon SON knockdown. Data are representative of n = 3 independent experiments. Error bars in all graphs represent standard deviation (SD) of tests. *p<0.05, **p<0.01. Statistical significance was determined by an unpaired two-tailed t-test. Source data are provided in the Source data file.

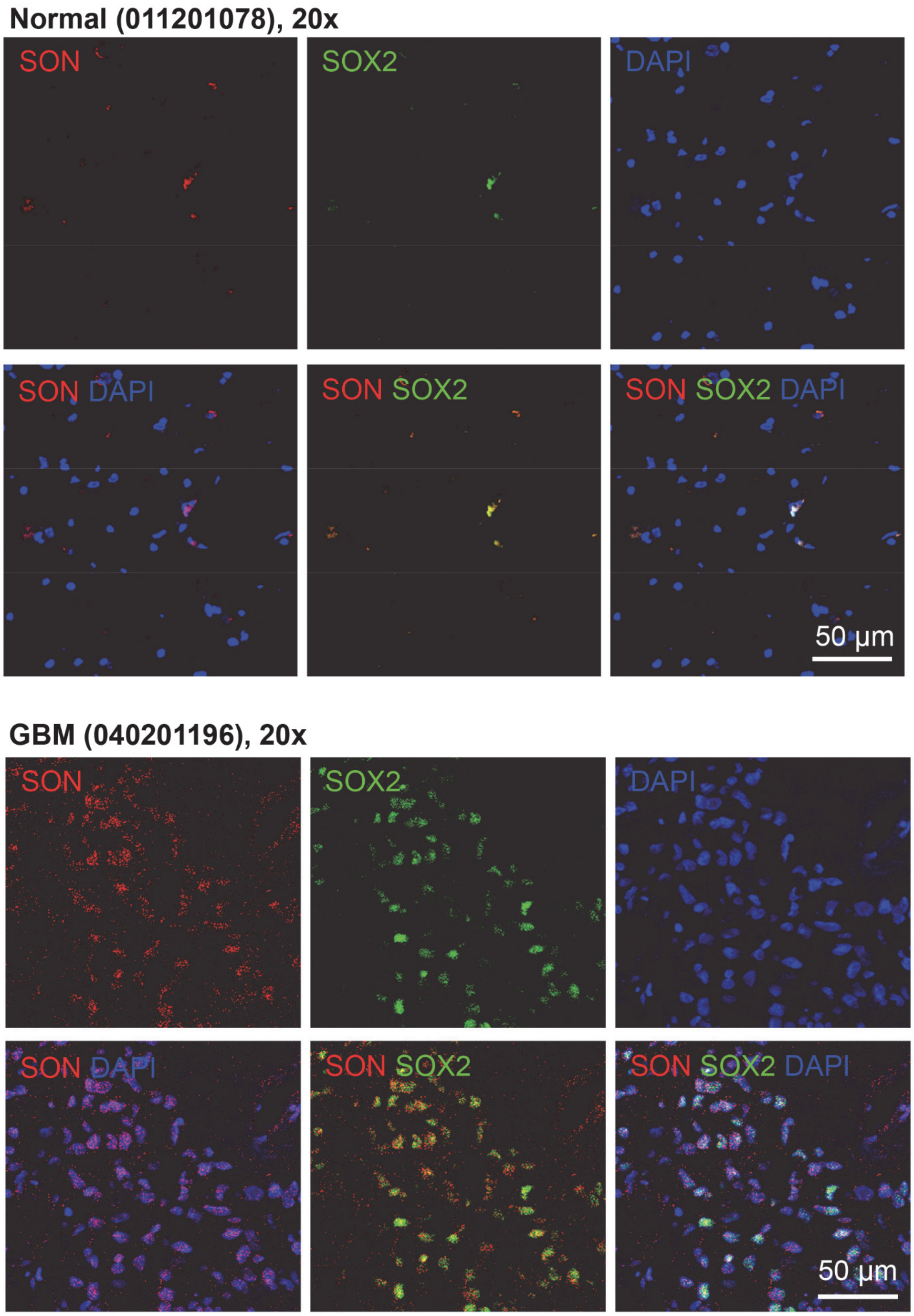

Supplementary Figure 4. SON and SOX2 are highly expressed in GBM patient samples. Immunofluorescence images were obtained from human glioblastoma (GBM) patients (040201196; Supplementary Table 1) and normal brain (011201078; Supplementary Table 1) from our cohort. Representative images of SON (red), SOX2 (green) and DAPI (blue) for GBM and normal brain were shown (n=3). Scale bar: 50 μm.

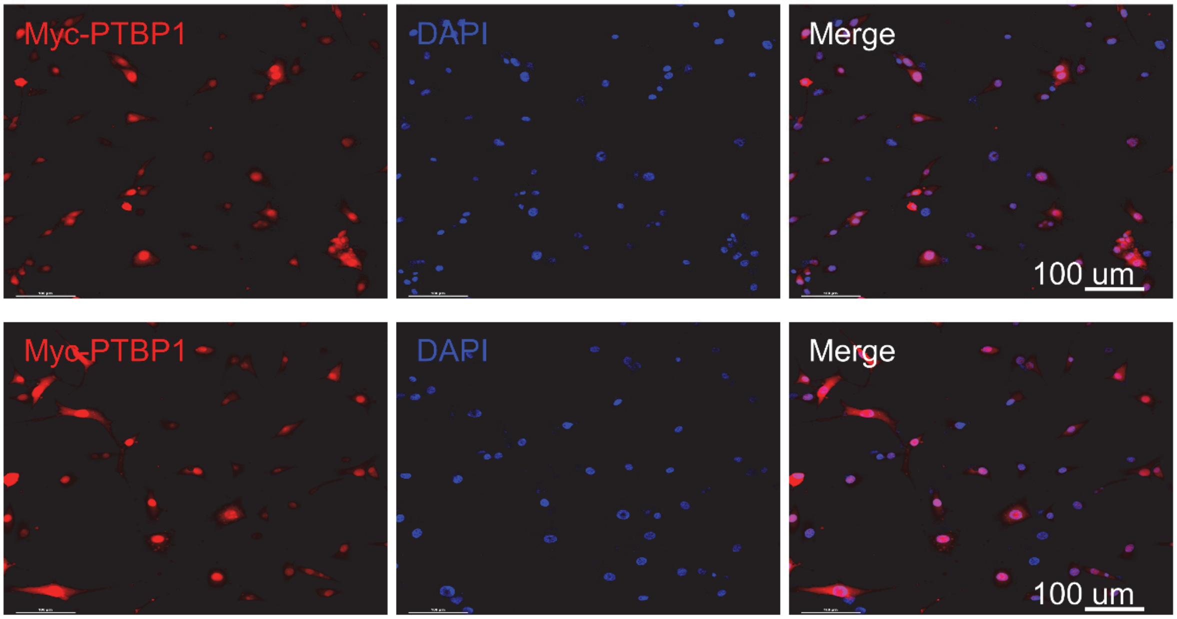

Supplementary Figure 5. Validation of transfection efficiency of the Myc-PTBP1 expression construct. U87MG cells were transfected with the pCMV-Myc-hPTBP1 (for human PTBP1) and harvested 48h after transfection for immunostaining with Myc antibody (for Myc-PTBP1) (using Myc antibody; red) and DAPI (blue) as well as for Western blots shown in **Figure 3g**. Images from two different transfections (top and bottom) were presented.

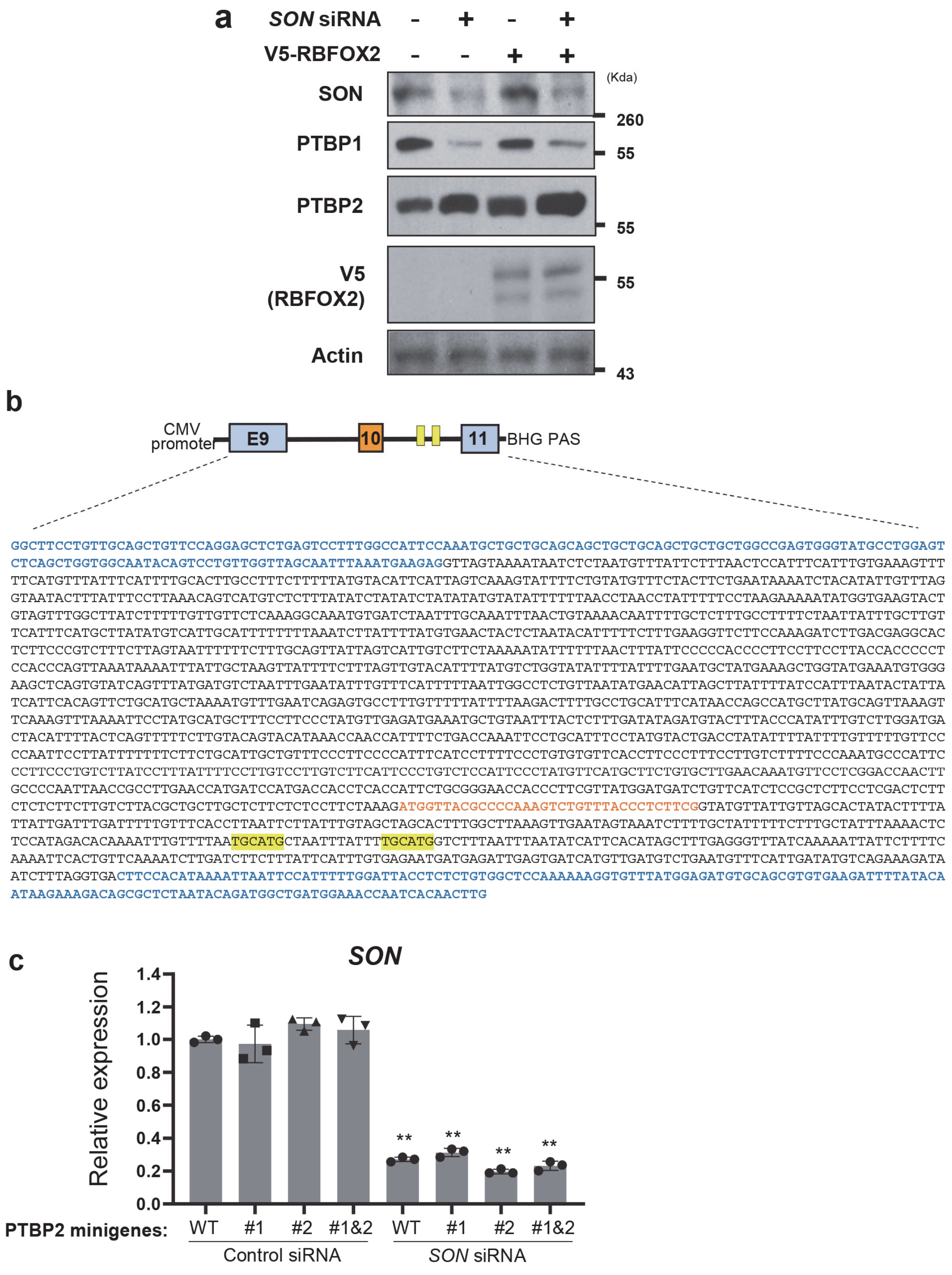

Supplementary Figure 6. RBFOX2 expression is required for SON knockdown-mediated PTBP2 upregulation. a. Overexpression of RBFOX2 (V5-RBFOX2) augmented SON knockdown-mediated PTBP2 upregulation while has no effect on SON knockdown-mediated PTBP1 downregulation. Cells were harvested 72 h after transfection (SON siRNA and

pcDNA3.1-V5-RBFOX2 as indicated) for Western blotting. Data are representative of n = 3 independent experiments. Actin, loading control. **b.** A schematic and the sequence of PTBP2 minigene. Genomic DNA sequence from exon 9 to exon 11 of human PTBP2 gene was cloned for the wild-type (WT) minigene. Two RBFOX2-bidning motifs identified within intron 10 were highlighted in yellow, and these motifs were altered as shown in Figure 4i to create Mut#1, Mut#2 and Mut#1+2 minigenes. **c.** qRT-PCR verifying knockdown in the cells used for minigene splicing assays (Figure 4j). Similar levels of SON knockdown were achieved by SON siRNA in the WT, Mut#1, Mut#2 and Mut#1+2 samples, ensuring that the different levels of minigene splicing are due to the modified RBFOX2 binding sites. The qPCR experiments were performed three times. Error bars in all graphs represent standard deviation (SD) of tests. N.S; not significant, *p<0.05, **p<0.01. Statistical significance was determined by an unpaired two-tailed t-test. Source data are provided in the Source data file.

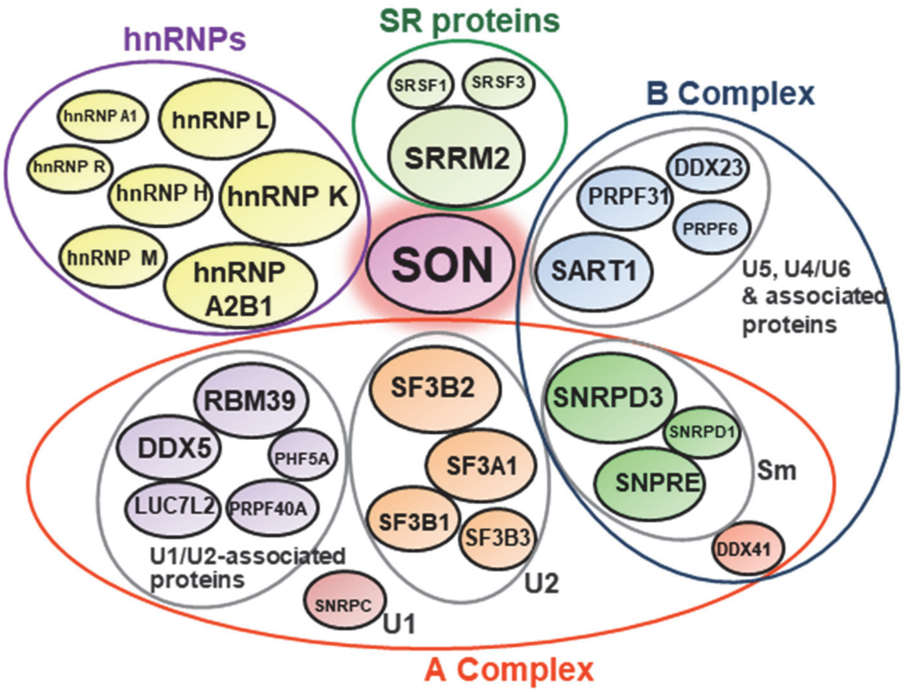

Supplementary Figure 7. Candidates of SON-interacting proteins identified by immunoprecipitation and mass spectrometry. The identified proteins are grouped into hnRNPs, SR proteins, and the spliceosome A-complex and B-complex. Increased circle size indicates higher number of abundance score of the specific protein.

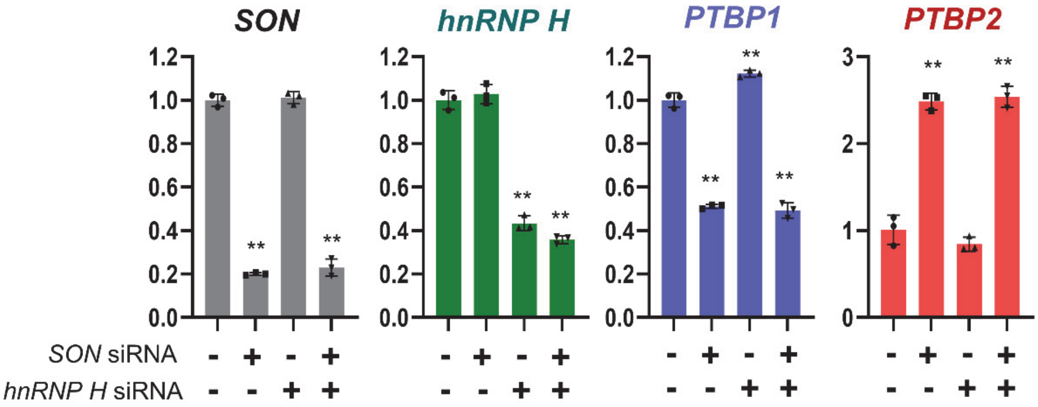

Supplementary Figure 8. hnRNP H does not regulate PTBP1 or PTBP2 expression.

The knockdown of hnRNP H (hnRNP H siRNA) does not change PTBP1 or PTBP2 expression. In addition, hnRNP H knockdown does not have any effect on SON siRNA-induced PTBP1 reduction and PTBP2 upregulation. hnRNP H siRNA was transfected on day 1, SON siRNA was transfected on day 2, and then cells were harvested for RNA isolation on day 4. The qPCR experiments were performed three times. Error bars in all graphs represent standard deviation (SD) of tests. N.S; not significant, *p<0.05, **p<0.01. Statistical significance was determined by an unpaired two-tailed t-test. Source data are provided in the Source data file.

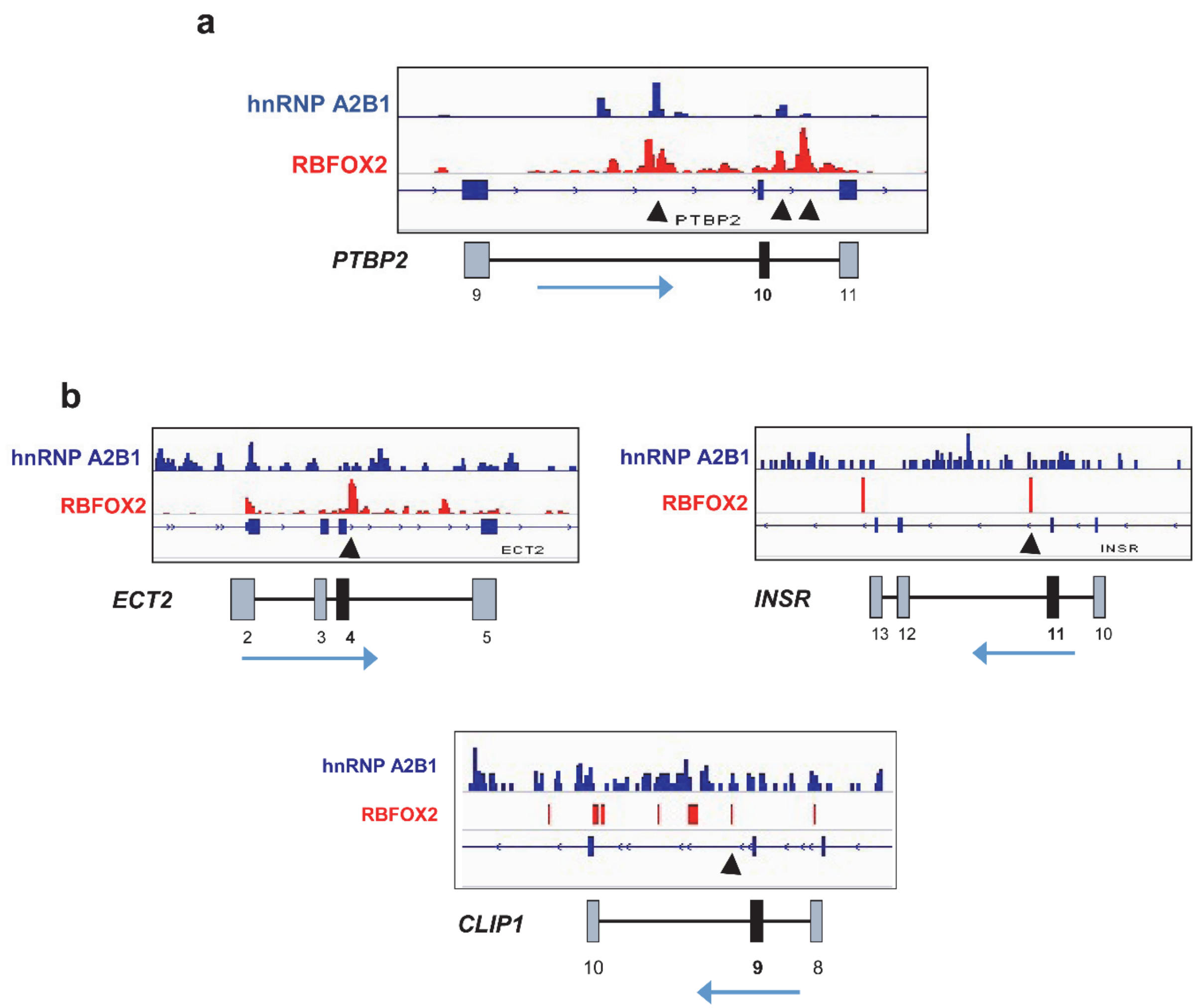

Supplementary Figure 9. The hnRNP A2B1 peaks overlap with the RBFOX2 peaks in several transcripts in which cassette exon inclusion is enhanced by SON knockdown.

a Integrative Genomics Viewer (IGV) images representing hnRNP A2B1 and RBFOX2 CLIP-seq in *PTBP2* mRNA. **b** Integrative Genomics Viewer (IGV) images representing hnRNP A2B1 and RBFOX2 CLIP-seq in *ECT2* (top left), *INSR* (top right), and *CLIP1* (bottom) mRNA. Schematics of mRNA structure of each gene and RBFOX2-targetted exon (black box) are indicated. The light blue arrows indicate the direction of the gene. The locations of potential RBFOX2 binding motifs are marked with black arrow heads.

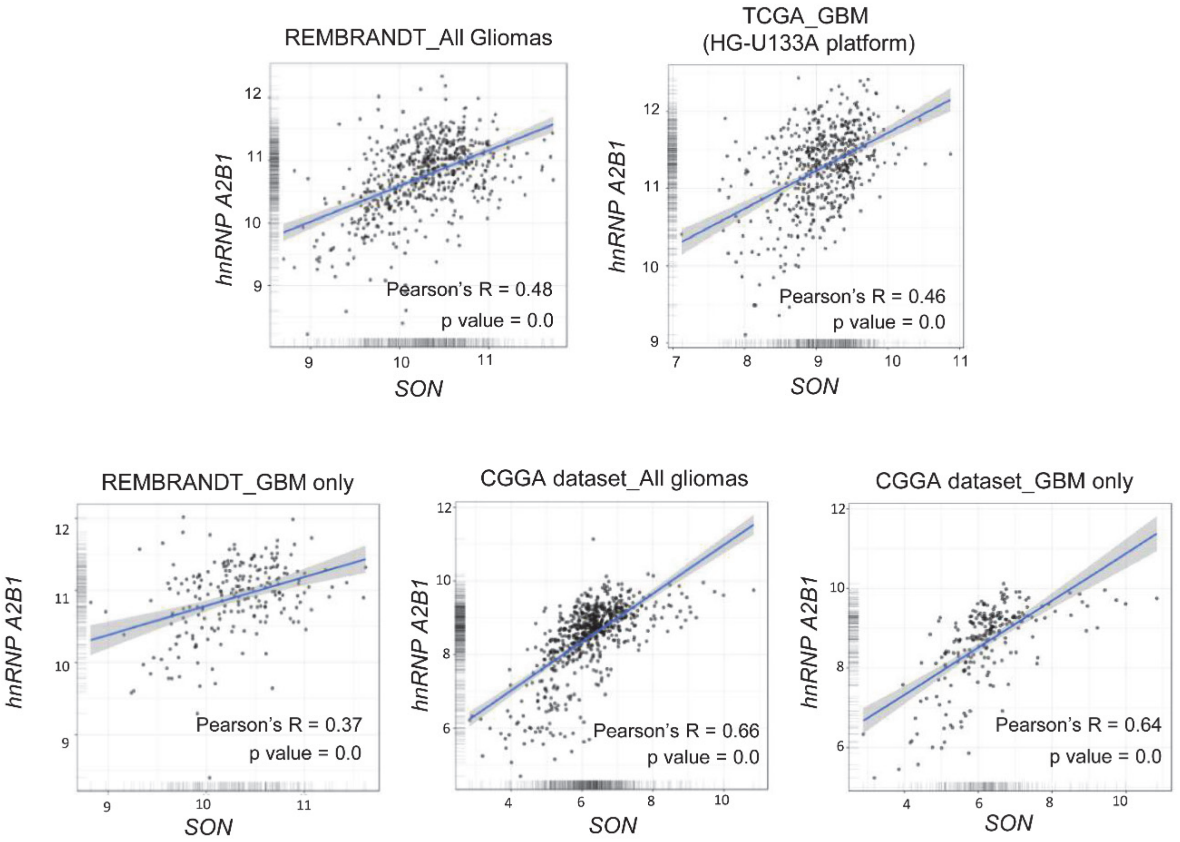

Supplementary Figure 10. Positive correlations between SON and hnRNP A2B1 mRNA expression patterns in brain tumor patient samples from the RAMBRANDT, TCGA and CGGA datasets. Correlations were analyzed using the GlioVis data portal. Solid blue line indicates line of best fit, with shaded areas depicting standard deviation (SD) confidence intervals.

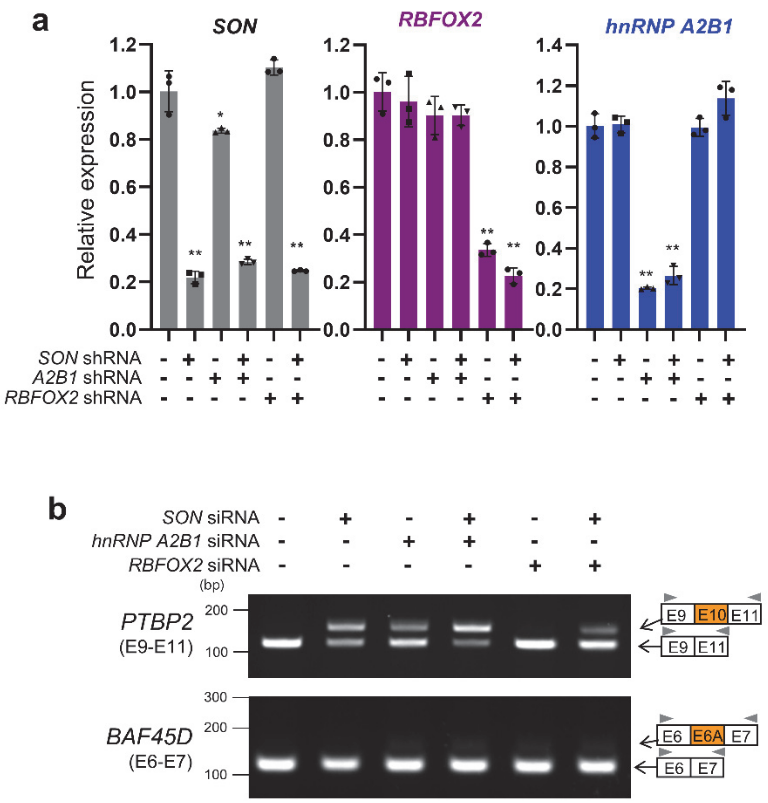

Supplementary Figure 11. Knockdown of SON, hnRNP A2B1, and RBFOX2 and their effects on alternative splicing of PTBP2 and BAF45D. a. Representative qRT-PCR data showing the levels of SON, RBFOX2 and hnRNP A2B1 upon shRNA-mediated knockdown of SON, RBFOX2 and/or hnRNP A2B1 in GSC#83 cells. RNAs were isolated 4 days after lentiviral infection of shRNA constructs. The qPCR experiments were performed three times. Error bars in all graphs represent standard deviation (SD) of tests. N.S; not significant, *p<0.05, **p<0.01. Statistical significance was determined by an unpaired two-tailed t-test. The data confirmed the efficiency of knockdown in each sample that were used for in vitro growth assays and sphere forming assays presented in Figure 7. **b.** RT-PCR analysis showing PTBP2 exon 10 inclusion/skipping in each condition of single- and combinational knockdown of SON, hnRNP A2B1 and RBFOX2 as indicated in U87MG cells. While PTBP2 exon 10 is regulated by SON, hnRNP A2B1 and RBFOX2, alternative splicing of BAF45D (exon 6A skipping/inclusion) was not regulated. Data are representative of n = 3 independent experiments. Source data are provided in the Source data file.

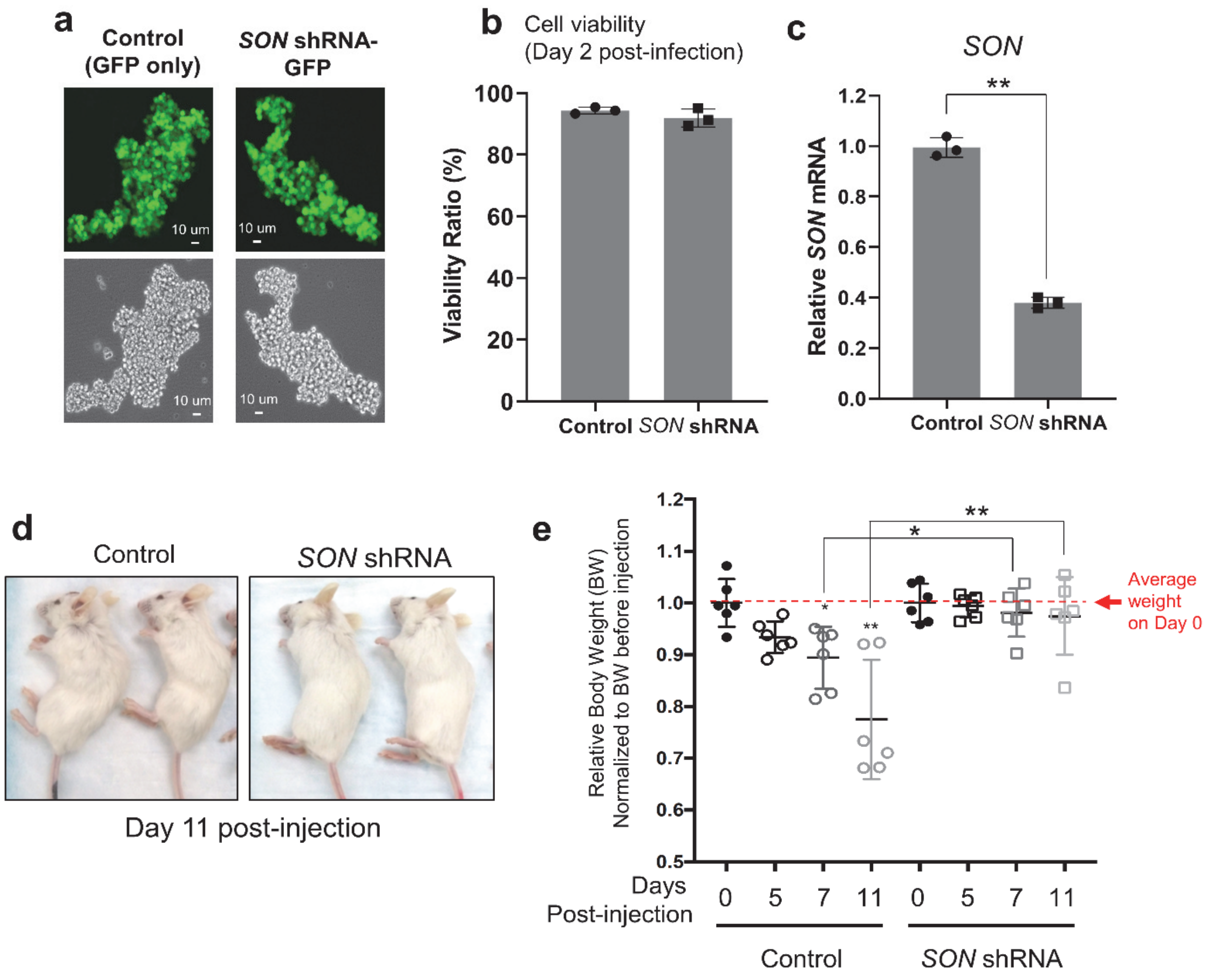

Supplementary Figure 12. SON knockdown in GSCs leads to inhibition of tumor growth in mice. a-c. GSC-83 cells (human, glioma stem cells) were transduced with a lentivirus expressing either control EGFP or SON shRNA. Cells were collected 2 days after lentiviral transduction, and injected into the mouse intracranially. EGFP expression (**a**), cell viability (n=3, Trypan Blue exclusion) (**b**), and relative SON mRNA level (n=3, normalized GAPDH) (**c**) in control and SON shRNA expressing cells at the time of injection are shown. Error bars in graphs (b and c) represent standard deviation (SD) of tests. Statistical significance was determined by an unpaired two-tailed t-test. **d**. Representative pictures of mice on day 11 post-injection from the control group with GBM symptoms (n=6, hunched posture and poor grooming) (left) or the SON shRNA group without GBM symptoms (n=6, right). **e**. Plot of relative body weight of the

mice after stereotaxic injection of GSC#83 cells transduced with a lentivirus expressing either EGFP or SON shRNA, normalized to the body weight of each mouse on the day of injection (n=6). *p<0.05, ***p <0.0001, 2-way ANOVA, Tukey's multiple comparisons test analysis by Prism7. Source data are provided in the Source data file.

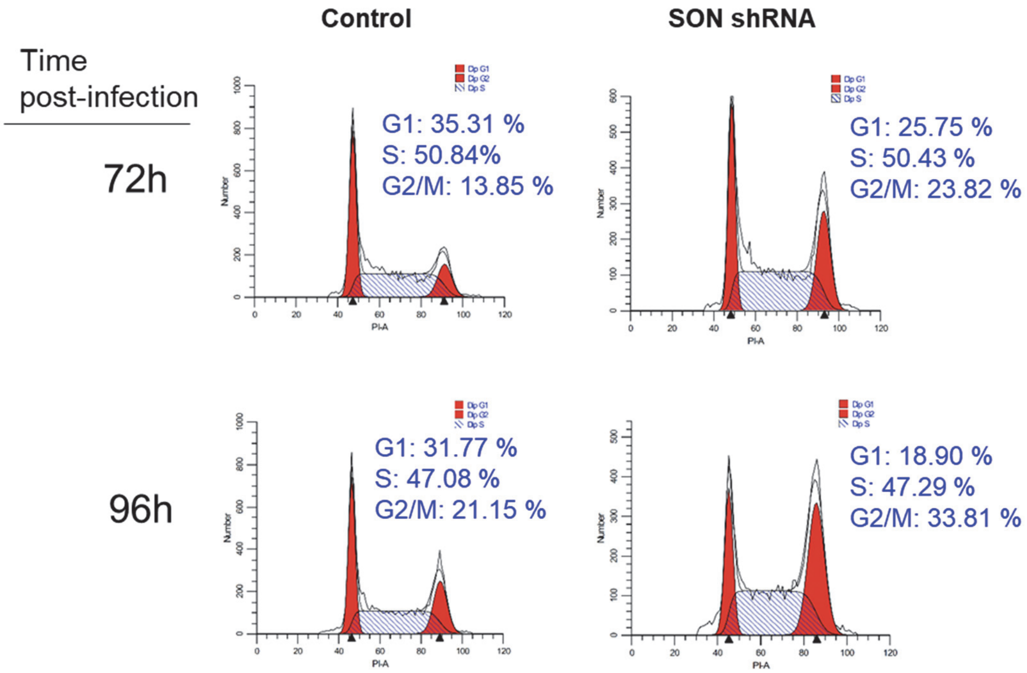

Supplementary Figure 13. SON knockdown in GSCs leads to G2/M cell cycle arrest. GSC#83 cells were transduced with lentivirus expressing either EGFP or SON shRNA. Part of the infected cells injected into mice were kept for continuous in vitro culture, and were collected at 72 or 96 hours after infection. Cell cycle was analyzed by PI staining followed by flow cytometry. Data are representative of n = 3 independent experiments. Source data are provided in the Source data file.

Supplementary Tables

Supplementary Table 1. Brain tumor patient sample information.

Code ID	Diagnosis	Age
001201088	Anaplastic Astrocytoma, Grade III	
010401033	Anaplastic Astrocytoma, Grade III	
030801105	Anaplastic Astrocytoma, Grade III	
030801108	Anaplastic Astrocytoma, Grade III	Range: 25-63
050101016	Anaplastic Astrocytoma, Grade III	
080901046	Anaplastic Astrocytoma, Grade III	Average: 40.5
090801030	Anaplastic Astrocytoma, Grade III	
101101058	Anaplastic Astrocytoma, Grade III	
110301019	Anaplastic Astrocytoma, Grade III	
111201089	Anaplastic Astrocytoma, Grade III	
030401050	Anaplastic Oligodendroglioma, Grade III	
030801107	Anaplastic Oligodendroglioma, Grade III	
041201292	Anaplastic Oligodendroglioma, Grade III	
050701082	Anaplastic Oligodendroglioma, Grade III	Range: 19-69
080401014	Anaplastic Oligodendroglioma, Grade III	
080601026	Anaplastic Oligodendroglioma, Grade III	Average: 40.5
090301015	Anaplastic Oligodendroglioma, Grade III	
110801066	Anaplastic Oligodendroglioma, Grade III	
110901074	Anaplastic Oligodendroglioma, Grade III	
150401020	Anaplastic Oligodendroglioma, Grade III	
020601051	Glioblastoma multiforme, Grade IV	
030501074	Glioblastoma multiforme, Grade IV	
030501083	Glioblastoma multiforme, Grade IV	
040201196	Glioblastoma multiforme, Grade IV	Range: 40-69
041001273	Glioblastoma multiforme, Grade IV	
050101009	Glioblastoma multiforme, Grade IV	Average: 57.3
050201028	Glioblastoma multiforme, Grade IV	
051101145	Glioblastoma multiforme, Grade IV	
060201014	Glioblastoma multiforme, Grade IV	
061201093	Glioblastoma multiforme, Grade IV	
000301025	Normal Brain (Astrogliosis)	
001001080	Normal Brain (Astrogliosis)	
010701059	Normal Brain (Astrogliosis)	
011201078	Normal Brain (Astrogliosis)	Range: 16-61
020701056	Normal Brain (Astrogliosis)	
020901085	Normal Brain (Astrogliosis)	Average: 43.6
030201011	Normal Brain (Astrogliosis)	
030501080	Normal Brain (Astrogliosis)	
040301210	Normal Brain (Astrogliosis)	
050301031	Normal Brain (Astrogliosis)	
070801067	Normal Brain (Astrogliosis)	

Supplementary Table 2. Primer sequences.

Name	Oligo sequence
- Primers for qPCR-	
F-SON Exon1 (total)	CAGATTTTTAGGTCTTTCGTGGT
R-SON Exon3 (total)	TTTTTCTGGAGCCCTCTTTC
F-SON Exon9 (SON F)	GCAATAAGGAACCCATCCTAGT
R-SON Exon12 (SON F)	AGGGCTCCATTTCTCAATACC
F-SON Exon3 (SON E)	GCTTCAGTATCTCACCAGTC
R-SON Exon5a (SON E)	GTTTGGCTTCTAAAACTCAG
R-SON Exon5 (SON B)	GCGATCTGTTTACATTTCTCAG
R-SON Exon7a (SON B)	GTTTAACCCGGCCCTCTG
F-GAPDH	GGCGCTGAGTACGTCGTGGAGTCCA
R-GAPDH	AAAGTTGTCATGGATGACCTTGG
F-PTBP1 Exon2-3	GTACAAAGCGGGGATCTGAC
R-PTBP1 Exon4	TTCCGGATGTGGATCACTCT
F-PTBP1 Exon6	GAGAACCTCTTCTACCCTGTGA
R-PTBP1 E6-int	CCTGTGCCCACAGCAATA
F-PBX1 (Exon5)	GAGGAAGCCAAAGAGGAGTTAG
R-PBX1 Exon6-7JCT	GGAAGAACCAGCCGAGTTG
R-PBX1 Exon6-8JCT	GTATCCACCAGCCGAGTTG
F-PTBP2 Exon13	GTAGCAGAAGAGGATCTACGAAC
R-PTBP2 Exon14	ACCCCCAATCTTCATCTTCCC
F-RBFOX2 Exon2	AACTCACCCAGCACACAAA
R-RBFOX2 Exon3	TAGAGACATGCAGCCGTTTC
F-hnRNP K	CAATGGTGAATTTGGTAAACGCC
R-hnRNP K	GTAGTCTGTACGGAGAGCCTTA
F-hnRNP A2B1	ACCAGCAACCTTCTAACTACG
R-hnRNP A2B1	GCCTCCTGGACCATAGTTTC
F-hnRNP M	GCTTTGAGCCATATGCCAATC
R-hnRNP M	CACGTATGTTACCTCACCAACT
F-hnRNP H	AATGGATTGGGTGTTGAAGC
R-hnRNP H	TATCCCATTTGGCACGATTT
F-PTBP2 Exon 8	CTGCATTGGACCCAGCTATT
R-PTBP2 Exon9-10JCT	GGGCGTAACCATCTCTTCATT
R-PTBP2 Exon9-11JCT	CATCTCCATAAACACCTCTTCATTT
F-INSR tot	CTGGTCTCCACCATTCGAGT
R-INSR-A	CGAGATGGCCTGGGGACG
R-INSR-B	CCTCGGCACCAGTGCCTG
F-ECT2 Exon2	CATCCACTACTGGGAGGACTA
R-ECT2 Exon4-5JCT	CAAAGCCCACTTTAATGTCCAT
R-ECT2 Exon3-5JCT	AAGCCCACTTTAATGTCCTTT
- Primers for RT-PCR-	
F-PTBP1 Exon6	GAGAACCTCTTCTACCCTGTGA
R-PTBP1 Exon7	GCCTGGAACTGGTTGTTCTT
F-PTBP1 Exon3	TTCTCTACTTGTGTCACTAACGG
R-PTBP1 Exon4	GCTGTCACCTTTGAACTTCTTG
F-PTBP1 Exon10-11	AACCTCAACCCAGAGAGAGT
R-PTBP1 Exon12	CTAGGGCGTTCTCCTTCTTATTG
F-PBX1 Exon6	CCGGTACAAGAAGAACATAGGTAA
R-PBX1 Exon8	CTGCGAGTCCATCACTGTATC
F-RTN4 Exon2	GCATCTGAGCCTGTGATACG
R-RTN4 Exon3	AGAGGAGACAGAGAAGGAAGAG
R-RTN4 Exon4	CAATGAAAGCAGCAGGAATAGG

F-TUBA1A Exon2	GGCAGTGTTTGTAGACTTGGA
R-TUBA1A Exon3	GCATCTTCTTTGCCTGTGATAAG
F-PTBP2 Exon9	AGCTGGTGGCAATACAGTCC
R-PTBP2 Exon11	CCATCAGCCATCTGTATTA
F-ECT2 Exon3	TTGGTTCAAGAAGCTGGAAAA
R-ECT2 Exon5	CAGAATCCTGAAAGTCCGTGA
F-KIF21A Exon22	GAAATAACCAGTGCTACCCAAAAC
R-KIF21A Exon24	GTTTAAAGGAGCATCCTCATCAGT
F-INSR Exon10	TGAGGATTACCTGCACAACG
R-INSR Exon12	GCTGGTCGAGGAAGTGTTG
F-CLIP1 Exon7	CATGTCCTGGAATTGGAAGC
R-CLIP1 Exon10	TGGAGTTTGTCAGCTTTGGTC

- CLIP- qPCR-

#1) F-PTBP2 Exon9	AGCTGGTGGCAATACAGTCC
#1) R-PTBP2 int9	AGAAAGGCAAGTGCAAAATG
#2) F-PTBP2 int9 bRB	TTGCTTGTTCATTTCATGCT
#2) R-PTBP2 int9 bRB	CTAAGAAAGACGGGAAGAGTG
#3) F-PTBP2 int9 1stRB	ATGTGGGAAGCTCAGTGTATC
#3) R-PTBP2 int9 1stRB	GCATGCAGAACTGTGAATGATAA
#4) F-PTBP2 int9 2ndRB	AGCCATGCTTATGCAGTTAAAG
#4) R-PTBP2 int9 2ndRB	CAGCATTTCATCTCAACATAGGG
#5) F-PTBP2 intron	CTTCGTTATGGATGATCTGTTC
#5) R-PTBP2 Exon10	AGACTTTGGGGCGTAACCAT
#6) F-PTBP2 Exon10	GTCTGTTTACCCTCTTCGGTATG
#6) R-PTBP2 intron10	GCCAAAGTGCTAGCTACAAATAAG
#7) F-PTBP2 int10RB	AGCACTTTGGCTTAAAGTTGAATAG
#7) R-PTBP2 int10RB	ACCCTCAAAGCTATGTGAATGA
#8) F-PTBP2 intron10	TACCTCTCTGTGGCTCCAA
#8) R-PTBP2 Exon11	CCATCAGCCATCTGTATTA

- Primer for DNA construct-

S-sh SON	TGCATTTGGCCCATCTGAGATTCAAGAGATCTCAGATGGGCCAAATGCTTTT TTC
AS-sh SON	TCGAGAAAAAAGCATTTGGCCCATCTGAGATCTCTTGAATCTCAG ATGGGCCAAATGCA
S-sh hnRNP K	TGATGTTTGATGACCGTCGCGTTCAAGAGACGCGACGGTCATCAAACATCT TTTTTC
AS-sh hnRNP K	TCGAGAAAAAAGATGTTTGATGACCGTCGCGTCTCTTGAACGCGACGGTCA TCAAACATCA
S-sh hnRNP M	TGCATCTTGTTGACATCGAATTTCAAGAGAATTCGATGTCAACAAGATGCTT TTTTC
AS-sh hnRNP M	TCGAGAAAAAAGCATCTTGTTGACATCGAATTCTCTTGAAATTCGATGTCAA CAAGATGCA
S-sh hnRNP A2B1	TGCTTCAGGTTATCGAAATAATTCAAGAGATTATTTCGATAACCTGAAGCTTT TTTC
AS-sh hnRNP A2B1	TCGAGAAAAAAGCTTCAGGTTATCGAAATAATCTCTTGAATTATTTCGATAAC CTGAAGCA
S-sh RBFOX2	TCACGTGTAATGACCAATAAGTTCAAGAGACTTATTGGTCATTACACGTGCT TTTTTC
AS-sh RBFOX2	TCGAGAAAAAAGCACGTGTAATGACCAATAAGTCTCTTGAACTTATTGGTCA TTACACGTGA
F- BamHI-KZ-V5-hRBFOX2	tttggatccGCCACCatgGGTAAGCCTATCCCTAACCCTCTCCTCGGTCTCGATTC TACGATGGAGAAAAAGAAAATGGTAACTCAGG
R- XhoI-hRBFOX2	tttctcgagTCAGTAGGGGGCAAATCGGCTGTAG
