# Supplementary material for: SON drives oncogenic RNA splicing in glioblastoma by regulating PTBP1/PTBP2 switching and RBFOX2 activity
Source: Nat Commun. 2021 Sep 21;12:5551. doi: 10.1038/s41467-021-25892-x (PMC8455679; doi:10.1038/s41467-021-25892-x)
Supplement: Supplementary file 3 — Description of Additional Supplementary Files [file 41467_2021_25892_MOESM3_ESM.pdf]

Description of Additional Supplementary Files

File Name: Supplementary Data 1
Description: SON-interacting proteins identified from SON-IP and LC-MS/MS
